# Comments on “Echo of extinction: the ivory-billed woodpecker's tragic legacy and its impact on scientific integrity”

**DOI:** 10.1093/biosci/biae141

**Published:** 2025-01-31

**Authors:** Michael D Collins

**Affiliations:** Naval Research Laboratory, Washington, DC, United States

In attempting to argue that the ivory-billed woodpecker (*Campephilus principalis*) is extinct, Michalak (2024) suggests that there has been a lack of scientific integrity but falls short of that standard by not addressing the strongest evidence for persistence. He tries to support his position with various flawed arguments, including an argument based on a map of reports of sightings of the pileated woodpecker (*Dryocopus pileatus*) that falls apart on close inspection. Michalak claims that, in order for the ivory-billed woodpecker to persist, its population must have exceeded 50,000 in the 1930s and must have recently numbered in the hundreds. It is easy to see that the first claim is false by considering that the whooping crane (*Grus americana*) persists even though its population was reduced to about 20 in the 1930s (McCoy 1966). If the other claim were true, any species would quickly go extinct after its population drops below the hundreds, but species such as the whooping crane have been known to persist in small numbers for decades.

During the past hundred years, the ivory-billed woodpecker has repeatedly been thought to be extinct only to be rediscovered. The announcement of the most recent rediscovery in Arkansas was the first report of this species by ornithologists in several decades (Fitzpatrick et al. 2005). Despite a report of sightings in Florida by another group of ornithologists the following year (Hill et al. 2006), the persistence of the species became controversial when neither group managed to obtain a clear photo. The strongest evidence that came out of those efforts is a series of sightings by numerous observers who were experienced at identifying birds, knowledgeable of the ivory-billed woodpecker, and acclimated to southern swamp forest habitats and the species that regularly occur in them. It is not plausible to dismiss as a series of mistakes all those sightings of a large bird that has distinctive and prominent field marks and remarkable flight characteristics.

Between 2006 and 2008, I obtained video footage to support three observations of birds that were identified in the field as ivory-billed woodpeckers on the basis of definitive field marks (Collins 2011, 2017). The initial analysis of the videos was later supplemented with additional analysis (Collins 2020, 2021, 2022, 2023). In order to facilitate reviewing the evidence, material that is distributed in a series of articles has been consolidated in an up-to-date summary (Collins 2024). Each of the videos contains stronger evidence than anything else that has been obtained during the past several decades. They show field marks, body proportions, flights, and other behaviors that are consistent with the ivory-billed woodpecker but no other species. The possibility of saving the ivory-billed woodpecker from extinction has been undermined by the lack of open discourse on this evidence.

## Summary of the strongest evidence

**Figure 1.**
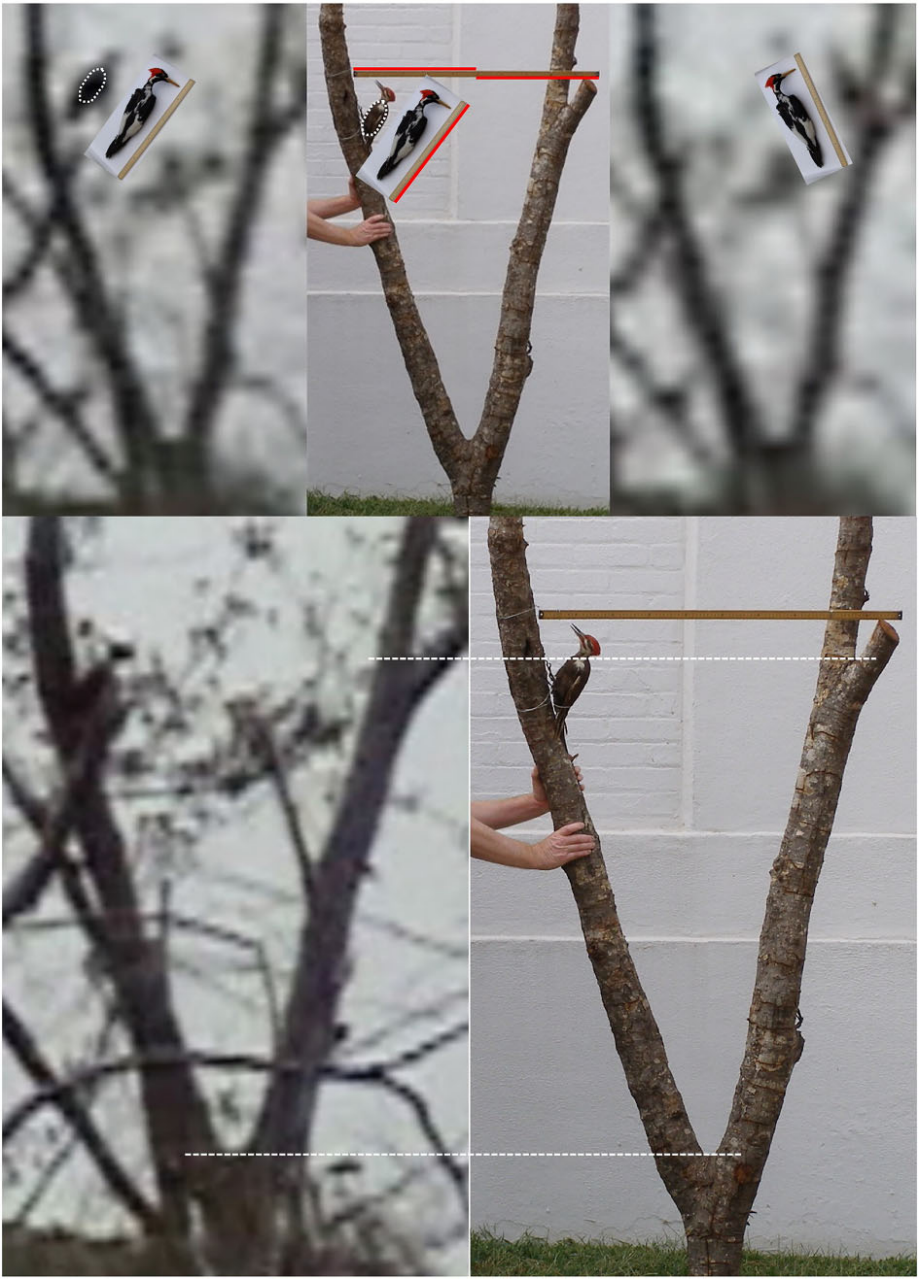
A comparison of the woodpecker in the 2006 video with specimens of the large woodpeckers. This version of the comparison (Collins 2022) is an improved version of a comparison that was published earlier (Collins 2017). As is indicated by the dashed lines, two forks in the tree were used to scale the images. The pileated woodpecker specimen was mounted on the tree specimen along with a meter stick. The ivory-billed woodpecker specimen was photographed with a half meter stick that was used for scaling. The woodpecker in the video was partially hidden by vegetation in the image on the lower left, but it was in full view during a short flight between limbs (top left). The outline of the body of the pileated woodpecker specimen is marked by a dashed curve that was copied without changing its size.

A video that was obtained in the Pearl River swamp in Louisiana in 2006 shows a large woodpecker perched on a tree with widely spaced forks that facilitated reliable scaling (the uncertainty in scaling is inversely proportional to the length of the scaling feature). The ivory-billed woodpecker is much more massive than the pileated woodpecker (the only other large woodpecker that exists north of the Rio Grande). As is shown in figure 1, the woodpecker in the 2006 video dwarfs a pileated woodpecker specimen and is comparable in size to an ivory-billed woodpecker specimen that is near the maximum size for that species. Its body would not fit through the largest opening of a pileated woodpecker cavity (Collins 2023). According to an avian artist who specializes in the ivory-billed woodpecker (one of her depictions of that species appears on the cover of the January 2006 issue of the *Auk*), the large woodpecker in the 2006 video has several behaviors and characteristics consistent with that species but not the pileated woodpecker (Collins 2011). The 2006 video was obtained during a flurry of activity along a concentrated stretch of English bayou during a five-day period, when I had five sightings with excellent views of definitive field marks and twice heard “kent” calls (once coming simultaneously from two directions).

A short distance up the same bayou in March 2008, I obtained video footage of a large bird that flew below my observation position. The bird in the 2008 video has the characteristic wing motion of a large woodpecker in which the wings are folded closed in the middle of each upstroke. The appearance of the reflection of the bird from the surface of the bayou made it possible to determine locations of the bird along its flight path, which were used to estimate the flight speed and determine that the wingspan is well over 24 inches. The two large woodpeckers are the only species of the region with that combination of wing motion and wingspan. An expert on woodpecker flight mechanics analyzed the wingtip motion and concluded that it is a large woodpecker (Collins 2011). Since the wingbeat frequency is about 10 standard deviations greater than the mean wingbeat frequency of the pileated woodpecker, the ivory-billed woodpecker is the only possibility, and the high aspect ratio of the wings, field marks, and flight speed are consistent with that species but not the pileated woodpecker. The 2008 video documents that I tracked the flight of the bird for about 10 seconds from an ideal position (at close range and nearly directly above) for observing the definitive dorsal field marks.

In January 2007, I visited an area in the Choctawhatchee River swamp in Florida, where a series of sightings had recently been reported (Hill et al. 2006). During an encounter with two distant ivory-billed woodpeckers that lasted for more than 20 minutes, I captured several events with a high-definition video camera. I observed definitive field marks and spectacular swooping flights consistent with an account of a landing with a “magnificent upward swoop” (Eckleberry 1961). The video shows several of the swooping flights, two takeoffs with deep and rapid wingbeats and loud “wooden” wing sounds that are consistent with an account by Tanner (1942), and an event involving a double knock (that is visible and audible) and other behaviors consistent with the ivory-billed woodpecker. In addition to the flights and other behaviors, the events in the 2007 video show field marks and body proportions consistent with the ivory-billed woodpecker.

## Elusiveness of the ivory-billed woodpecker

**Figure 2.**
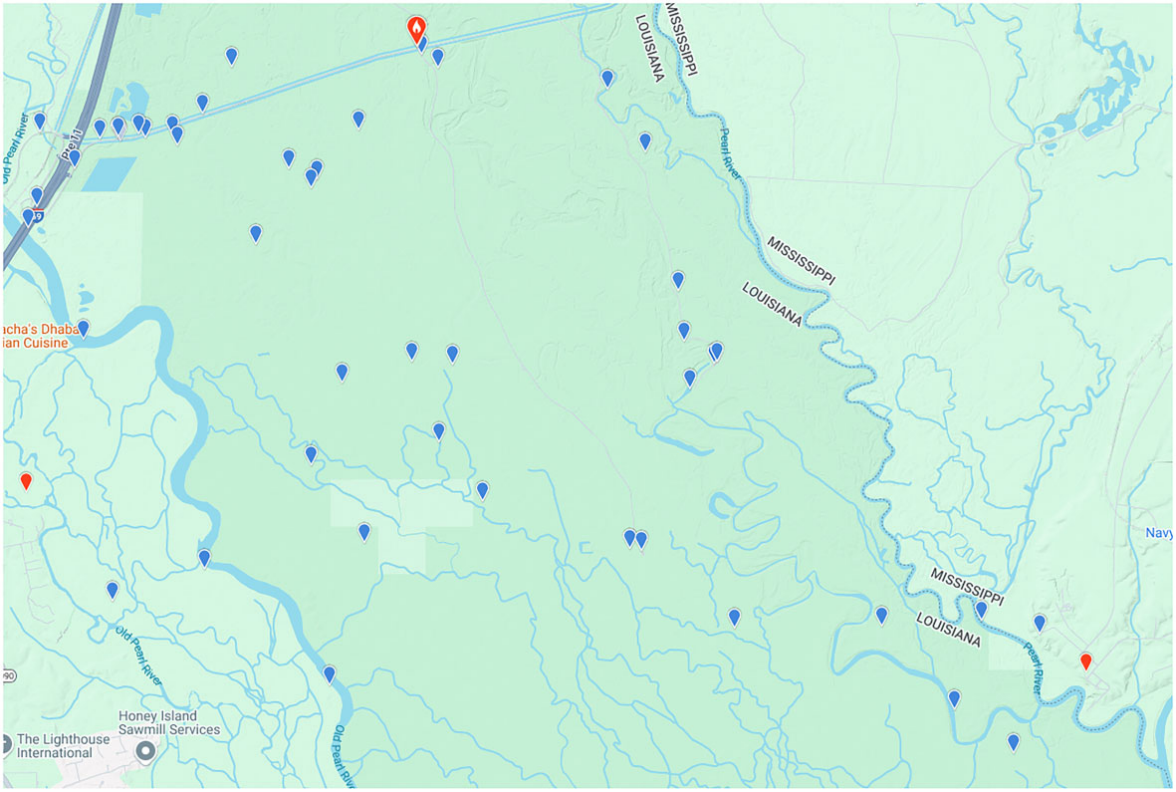
Distribution of eBird reports of pileated woodpeckers in a region of the Pearl River swamp that is well to the north of Interstate Highway 10. Image: Courtesy of the Macaulay Library and eBird.

**Figure 3.**
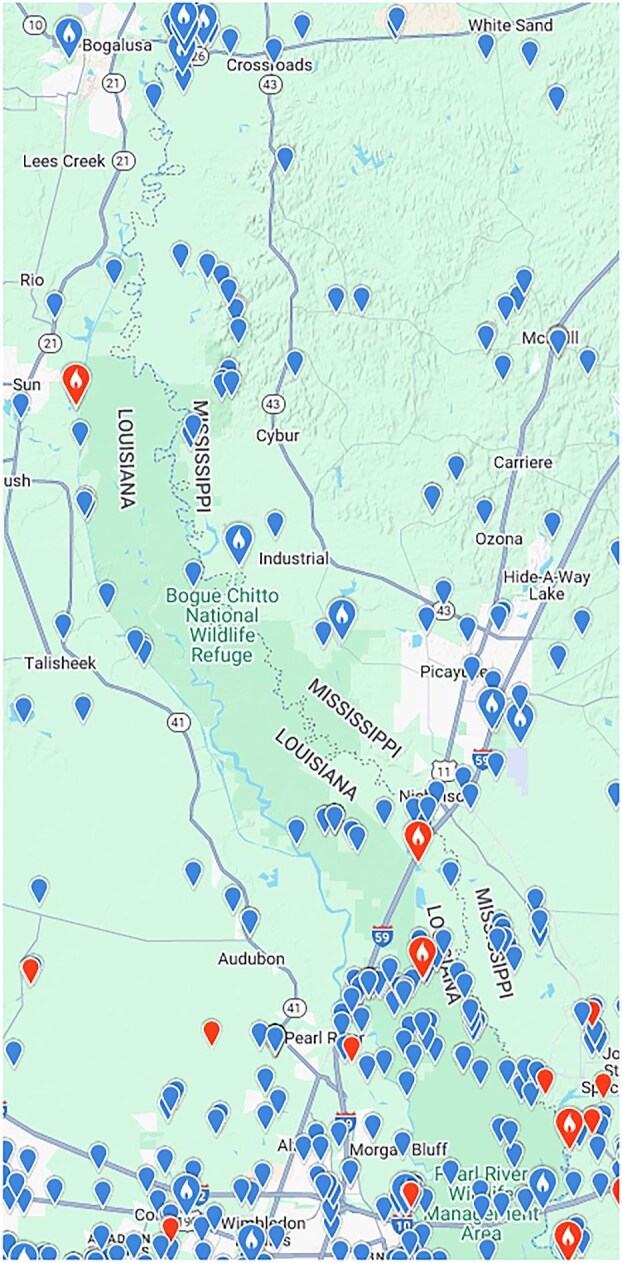
Distribution of eBird reports of pileated woodpeckers in a wider region of the Pearl River swamp. Image: Courtesy of the Macaulay Library and eBird.

Michalak discusses a map of eBird reports of pileated woodpeckers in the Pearl River that does not contain any information relevant to the question of the persistence of the ivory-billed woodpecker. The cypress-tupelo swamp in the lower two-thirds of the area shown in that map lacks the more diverse forest that is believed to be preferred by ivory-billed woodpeckers. The focus of my study in the Pearl River is the area appearing in figure 2, which is located to the north of the cypress-tupelo zone, to the south of Old Highway 11, and in the interior of the darker green area between the main channels of the Pearl River. Many of the eBird reports in that area were from on or near Old Highway 11, Oil Well Road, and Indian Bayou Road. In the remote areas away from roads, there were only 16 reports, and 12 of them were from Cornell's Mobile Search Team (Banfield, Lammertink, McCafferty, and Setiorini), who visited the area to search for ivory-billed woodpeckers in February 2007. Only four of the reports (from only three different observers) are suggestive of activity by casual bird watchers, and three of those are from a brief period in February 2008. This is consistent with the fact that, during 8 years of field work, I never saw bird watchers in remote areas away from roads. Appearing in figure 3 are eBird reports for a larger area in the Pearl River. To the north of Interstate Highway 59, there were only a few reports from the interior of the dark green area, and most of those reports were from on or near roads. As was discussed in a previous article (Collins 2019), the fact that bird watchers rarely visit remote areas in southern swamp forests is only one of several factors that account for the ivory-billed woodpecker's remarkable history of elusiveness.

## Consequences

Michalak is not alone in pushing the narrative that the ivory-billed woodpecker is extinct without addressing the strongest evidence (Collins 2019). There are likely to be consequences if the lack of an open discourse involving the most relevant information continues to prevent the truth from coming to light. The whooping crane, California condor (*Gymnogyps californianus*), and Kirtland's warbler (*Setophaga kirtlandii*) would likely be extinct by now if not for conservation programs that were established more than 50 years ago. There has never existed such a program for the long-neglected ivory-billed woodpecker.

## Author Biography

Michael D. Collins (michael.d.collins118.civ@us.navy.mil) is affiliated with the Naval Research Laboratory, in Washington, DC, in the United States.
